# Cats in a Cat Café: Individual Cat Behavior and Interactions with Humans

**DOI:** 10.3390/ani15223233

**Published:** 2025-11-07

**Authors:** Elin N. Hirsch, Belén Navarro Rivero, Maria Andersson

**Affiliations:** Department of Applied Animal Science and Welfare, Swedish University of Agricultural Sciences, P.O. Box 234, 53223 Skara, Sweden; elin.hirsch@slu.se (E.N.H.); belen.nariv@gmail.com (B.N.R.)

**Keywords:** cat behavior, cat café, cat welfare, human-cat interaction

## Abstract

**Simple Summary:**

Cat cafés, where visitors can spend time with cats and even adopt them, are becoming more common. While these cafés are enjoyable for people, it is less clear how they affect the cats that live there. In this study, we observed 27 cats living in a Swedish cat café to understand how they utilized the space, interacted with one another, and responded to human visitors. We found that cats often preferred elevated places, such as shelves or cat trees, and quiet hiding spots, especially when there were many customers in the café. Interactions between cats were rare and usually friendly, while cats’ responses to people varied from seeking contact to choosing to rest out of reach. These findings indicate that cats in cafés require sufficient space to allow them to choose whether to interact or withdraw. Designing cat cafés with vertical structures, separate rooms unavailable for customers, and safe hiding places will help reduce stress, support natural behaviors, and improve cat welfare. This knowledge is important for café owners, animal welfare organizations, and future adopters, as it ensures that cats have positive experiences before finding permanent homes.

**Abstract:**

Cat cafés are gaining popularity worldwide, yet little is known about their impact on feline welfare. This study examined a Swedish cat café from an animal perspective by assessing space use, cat–cat interactions, and cat–human interactions. A total of 27 neutered domestic cats (12 males, 15 females), with a median stay of 8 weeks, were directly observed in groups of 8–9 individuals for a total of 227 h over 70 days. Scan sampling and focal observations were conducted without prior knowledge of cat background to minimize bias. Results showed that elevated structures (χ^2^-goodness-of-fit test, χ^2^ (2) = 1234.2, *p* < 0.001, post hoc Bonferroni correction *p*_adj_ = 0.013) were important, particularly during periods of high customer occupancy, when cats preferred vertical levels or cat-only rooms. Cat–cat interactions were infrequent (0.58 interactions/cat/h) and mostly affiliative (51.9%), consistent with avoidance as a conflict-reduction strategy. Cat–human interactions were absent in 44.4% of the observation time. Cats varied in their responses to human presence, from engaging to avoiding. Findings suggest that cat cafés should provide complex vertical environments, hiding places, and private areas to support natural behaviors, reduce stress, and promote group stability. As cats come into contact with large numbers of visitors prior to adoption, further research should assess its long-term effects on cat–human relationships and optimize café design to balance cat welfare and visitor experience.

## 1. Introduction

Driven by an increasing need for stress resilience in Japanese society, the first animal cafés opened 20 years ago [1]. In Sweden, the first cat café opened in 2019 in Stockholm. Cat cafés are not only popular businesses to visit but also provide a new opportunity to explore the human–cat relationship and the behavior of domestic cats (*Felis silvestris catus*) in unfamiliar environments, together with unfamiliar humans.

Despite conflicting results as to the benefit of keeping pets [2], referred to as the “pet effect” [3], many people find comfort when interacting with animals. A meta-analysis on pets’ influence on human physical and mental health showed that pet guardians are more active and experience better mental well-being, such as enriched mood, increased happiness, and life satisfaction [4]. Earlier studies show that living with a pet can provide several benefits, such as mitigating depressive symptoms [5,6,7], decreasing blood pressure when facing a stressor [8], and even reducing the risk of dying from cardiovascular diseases [9]. There are also studies indicating improved emotional health, helping people suffering from depression, anger, or isolation [3,6,7]. The unconditional acceptance of pets can explain this phenomenon. Similarly, cat café guests often describe their experience of interacting with cats as less demanding and less stressful than interacting with other humans [1]. At the same time, there are indications that interaction with humans can have a positive influence on cat behavior and welfare [10], and that handled shelter cats were more likely to remain or to be scored as content, have improved immune function, and reduced incidence of upper respiratory tract disease [10,11]. Similarly, approximately half of the tested socialized shelter and privately owned cats actively selected interactions with humans in a free-operant preference assessment [12].

However, cats can be vulnerable in the café environment. Their solitary origin and late exposure to artificial selection may hinder their ability to cope with many of the stressful situations imposed by humans [13]. These include, but are not limited to, cohabitation with unfamiliar cats and/or confinement. Domestic cats are descended from the solitary and territorial predator, the African wildcat (*Felis silvestris lybica*), partly through a self-selecting process, in contrast to most other domestic animals [14].

Today, as a facultative social animal, the domestic cat shows extensive flexibility in social organization, ranging from solitary living to stable, matrilineal groups when food resources are sufficient [15]. Although cats are solitary hunters, primarily related females may cooperate in rearing their young [16]. Unlike other territorial animals, domestic cats lack conflict-minimizing signals to preserve group stability [13]. Instead, they rely on keeping a distance from unfamiliar conspecifics and dispersal in case of conflict, which is not always possible in human-made environments [17,18]. High cat density [19] and cohabitation with non-socialized individuals can increase signs of stress within a group [20]. Affiliative behaviors and proximity enhance group cohesion, particularly among relatives and familiar individuals [21,22,23].

Still, the adaptation of domestic cats to a new environment can be an exceptionally complex and prolonged process. Research in shelter settings shows that most cats adapt within two weeks; yet approximately 4% do not exhibit a drop in behavioral stress during this time [24]. For some group-housed shelter cats, a decrease in behavioral stress indicators (i.e., abnormal sleep patterns and decreased maintenance behaviors) can take over eight months [25]. Stressed cats usually exhibit suppressed natural behaviors, such as exploration, play [26], and maintenance behaviors (e.g., grooming, eating, and elimination) [27]. In the event of chronic stress, cats are likely to become inactive and passive [26,27]. Behavioral stress scores seem to increase with age and depend on previous experiences [27,28], such as social experiences [20].

The set-up of the environment influences behavior and signs of stress. Providing space to keep a distance from other conspecifics allows cats to live in relatively large densities [29], especially when concealed areas and elevated vantage points are provided [27]. Therefore, providing complex tridimensional spaces reduces stress levels and aggression while promoting natural behaviors and coping strategies (i.e., hiding) [30,31]. The ability to cope with stressors not only improves cat welfare and health but may also increase their adoption chances [18,28,30].

A challenging aspect of cat cafés is the combination of high human density, unfamiliar humans, and unpredictable handling, well-known stressors for domestic cats according to research in lab facilities and homes [26,32]. Lichtsteiner and Turner [32] found that the basal urinary cortisol levels of privately owned cats were affected by total available space (m^2^) and the number of persons per household. They also suggest that the effect of human density might be influenced by noise or human composition (i.e., children, employed adults, etc.) [32].

While several studies provide valuable knowledge about the human–cat relationship, there is still much to discover, particularly in the café environment. Socialization towards humans (2–7 weeks) is essential for the social development of kittens [33], even if their behavior towards humans continues to develop until approximately 4 months of age [34]. Cat sociability increases with human attentional state and depends on cat origin: shelter cats spend the most time near humans, even if these are unfamiliar or inattentive [35].

One influential component of the human–cat relationship is the cat and human personality [36]. While individuality determines cat behavior during first encounters with humans, human behavior seems to depend on age and gender [37]. Differences in vocalization, distance regulation, and posture among women, men, and children influence human–cat interactions [37,38]. Children usually initiate interactions by approaching the cats immediately, whereas adults (especially women) prefer to vocalize first [37]. Studies in the home also indicate that humans tend to start interactions [38], but that these last longer when initiated by a cat [39]. A recent study of the behavior of 12 cats in a cat café in France [40] showed that cats displayed a clear preference for visual and bimodal cues (visual and vocal cues expressed at the same time) addressed by non-familiar humans compared to vocal cues only.

The objective of this study was to describe a cat café from an animal perspective and map individual cat behavior by (1) investigating how cats utilize the available space and resources provided and (2) studying the social interactions between resident cats, as well as between resident cats and humans. As the café offers many areas to promote the expression of cat-typical behaviors (e.g., moving in three-dimensional space) and coping strategies (e.g., hiding), we expect that most cats prefer concealed resting spots (e.g., cat beds) or raised points (e.g., shelves, cat trees, etc.). At periods of high customer occupancy, we expect that cats would spend more time out of the reach of visitors or engage in stress-related behaviors, including increased inactivity and decreased exploration and maintenance. Domestic cats avoid conflict and competition by maintaining distance from conspecifics as well as concealing their presence. Thus, we predict that few cat–cat interactions will be observed, except for affiliative interactions between related or familiar cats. Since age and gender affect the behavior of the human partner and thereby influence cat–human interactions (see, e.g., [41]), we expected to observe similar differences in a cat café environment.

## 2. Materials and Methods

### 2.1. Study Site

The study took place at a cat café located in Stockholm (Sweden). Observations were conducted in two periods, October–November 2020 (Period 1) and January–April 2021 (Period 2). The cat café was open daily during the first period from 10:00 to 20:00. However, due to the response to the COVID-19 pandemic, in the form of restrictions and bans, primarily against larger gatherings and keeping social distances, several schedule changes occurred throughout the second period. During this time, the most common opening hours were weekdays from 12:00 to 18:00 and weekends from 10:00 to 20:00.

**Figure 1:**
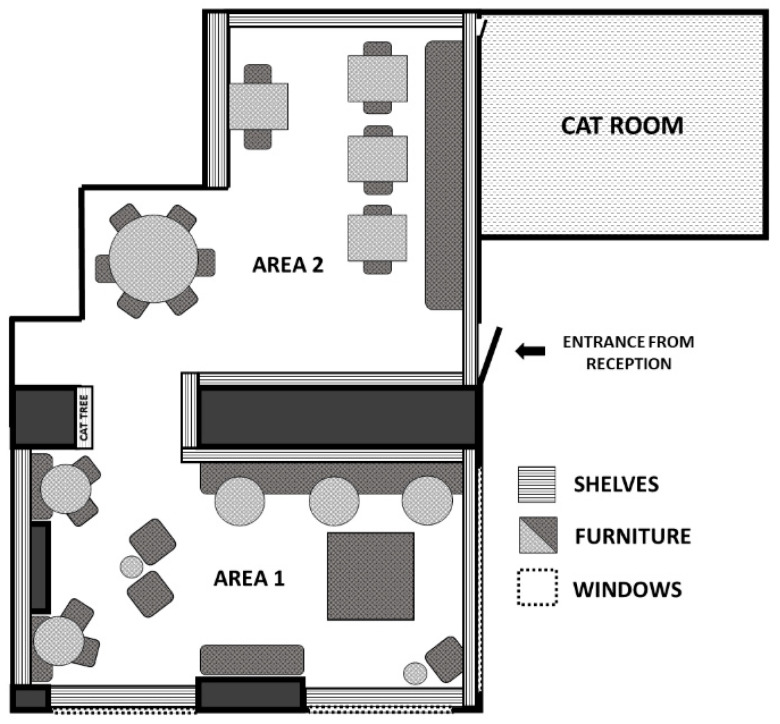
Representation of the cat café and areas available to the cats. The lounge and the cat room were 60 and 18 m^2^, respectively.

The site comprised three main areas: the cat room (18 m^2^), the cat café lounge (60 m^2^), and a reception (not accessible for cats) through which all customers entered after registration (Figure 1). The cat room was accessible only to the staff and the cats and accommodated their carriers, beds, and litter boxes. All interactions with customers occurred in the lounge.

The cat café staff comprised 10 members. There were two staff members present on weekdays and three during weekends. Staff were normally present beyond opening hours, and attended to the care of the cats (e.g., nail clipping, medication, teeth brushing, etc.) and cleaning routines. Cleaning took place on Tuesdays, Fridays (lounge), and Wednesdays (cat room). Additionally, the space was vacuum cleaned every day before opening times.

No children under the age of 10 were allowed in the cat lounge. Customers could book visits either in advance or upon arrival. A maximum of 14 visitors were allowed at any time. Before entering the lounge, customers registered and paid at the reception, where food and beverages could be ordered. The staff introduced the café rules to the customers before admission. In summary, these rules specified that all interactions occur on the cats’ terms: cats could not be held, followed, or forced to stay. Playing had to take place with the toys made available by the café, and feeding the cats was strictly prohibited. Additionally, following the recommendations from the Public Health Agency of Sweden in response to the COVID-19 pandemic, no more than four visitors were allowed in the reception at any given time. Staff also reminded customers to remain seated if possible and to keep their distance from other guests.

Cats were provided with water ad libitum in three water bowls and one cat fountain in the lounge. Food was provided by the staff four times per day, and litter boxes were cleaned twice a day. Feeding occurred at approximately 8:30, 12:00, 15:30, and 19:30 during the first period of the study. These times were modified during weekdays in the second period to accommodate the changes in opening hours (i.e., 10:30, 14:30, 17:30, and 19:30). However, feeding times remained unchanged during the weekends of the second period. Customers were provided with the opportunity to feed the cats snacks provided by the staff during the day.

### 2.2. Subjects

A total of 27 domestic cats, all available for adoption, participated in the study, 12 males and 15 females. The age of the cats ranged from <1 to 10 years, with a median of 4 years (IQR = 1–5.5). The age of formerly stray cats was estimated by a veterinarian. All cats were neutered/spayed and assessed by a veterinarian upon arrival. Cats came from a non-profit organization that relocates dogs and cats to foster homes in Sweden. The cat café often selects cats based on temperament and age (usually younger than 3 years old). However, the café was also open to old or poorly socialized cats. The cat groups were set to a maximum of 9 individuals (max = 9, min = 8) but were allowed to fluctuate due to adoptions and the arrival of new cats. Adoption was carefully monitored by the chairperson of the non-profit foster organization together with the cat café manager and comprised applications, interviews, and visits to the cat café.

The observer refrained from reading any background information about the cats until the completion of observations, to minimize bias. Background information included socialization status, origin, and kinship (Table 1). Of the subjects, 7 were originally stray cats, 2 colony cats, and 18 formerly privately owned cats.

According to the cat café standards, incoming cats should stay between 6 and 24 weeks. The median stay was 8 weeks (IQR = 6.75–10).

### 2.3. Data Collection

This study was based on 227 h of direct behavioral observations collected over 70 days. All observations were performed by the same observer. Scan samples and focal observations of human–cat interactions were recorded using the Behavioral Observation Research Interactive Software (BORIS), v 7.9.6 [43]. Cat–cat interactions were noted in a notebook due to their low occurrence. Each session was preceded by a 10 min habituation period to account for the effect of the observer’s presence, who avoided any kind of interaction with the cats. The observer decided from where the observation should take place before each session, based on where the customers were seated, to minimize interference. The recommendations from the Public Health Agency of Sweden, regarding the COVID-19 pandemic, were also considered in the choice of observation position (i.e., social distancing from customers). The observer switched positions in exceptional circumstances (e.g., high customer occupancy). These times were noted in detail.

#### 2.3.1. Period 1

During the first period of the study (October–November 2020), observations occurred daily in two 2 h sessions (morning and afternoon) for five days per week, including the weekends. The observation time was assigned beforehand using pseudo-randomization: morning session (10:00–12:00 or 12:00–14:00) and afternoon session (14:00–16:00, 16:00–18:00, or 18:00–20:00). These time intervals were chosen to consider and include all opening hours of the cat café (10:00– 20:00) and capture the potential effect of high customer occupancy.

#### 2.3.2. Period 2

The observation times during the second period (January–April 2021) needed to be adjusted due to changes in opening hours. During this period, observations occurred in one 2 h session on weekdays (12:00–18:00) and for two 2 h sessions on weekends (10:00–20:00) for five days per week. No observations occurred from 18:00 to 20:00 during this period since the cat café was closed during this time, for most of the week. The same pseudo-randomized methodology as described previously was used to choose the observation time.

#### 2.3.3. Behavioral Observations

To determine the occupancy level of the cat café, the observer noted the number of visitors and cats in the lounge at the time of observation. Additionally, the observer collected the number of daily bookings (customers per day) every week.

Scan samples were performed every 10 min, to record the location and behavior of the cats during each 2 h session. The observer classified the cat café lounge into two main areas (“Area 1” and “Area 2”) and three vertical levels (“floor”, “furniture”, and “shelf”) to determine the location of each cat (e.g., “Area 1, shelf”) (Figure 1). The ethogram used to record the behaviors of cats was based on the standardized ethogram for *Felidae* from [44] (Table S1). If a cat was absent from the lounge, it was noted by the observer as “out of sight” along with a modifier (“Cat room”).

Cat–cat interactions were assessed using an all-occurrence methodology, since only a few social interactions were observed during the pilot study. These interactions were classified as agonistic, affiliative, or ambiguous, based on the classification from [44,45,46] (Table S2). The observer also considered the subjects involved, their sex, the interaction type (contact or non-contact interaction), and proximity (<40 cm or >40 cm).

Focal observations were performed once per day in 15 min periods to record the interactions between cats and customers. Additionally, the observer adhered to the same scanning methodology as described for space use. If the group comprised nine cats instead of eight, the observation time was extended by 15 min (i.e., each observation lasted 2 h and 15 min) to include enough time for the focal observations of all individuals. Additionally, an extra group scan was performed. The observation order of each cat was randomized in advance to avoid bias.

Cat–customer interactions were grouped into the following categories: contact attention, non-contact attention, no attention, and no interaction, based on the classification from [36,44,46,47] (Table S3). The observer noted the sex of the cat, the customer’s gender and approximate age (youth or adult), and the area and vertical level in which the interaction occurred. Additionally, interactions between the cats and the staff were also noted but not included in the analysis of human–cat interactions.

### 2.4. Data Analysis

Statistical analysis was performed using RStudio, v 1.4.1106 [48] and figures were produced using the package ggplot2, v 3.3.3 [49]. Before analysis, the variable customer occupancy was converted into a factor with three levels (i.e., low: <21 guests; med: 21–29 guests; high: >29 guests). Similarly, the study cats were classified according to their stay period in the cat café into the following categories: <6 weeks, 6–12 weeks, and >12 weeks. These criteria were derived from the cat café standards and Swedish legislation [50] about the temporary housing of domestic cats. Potential departures from expected distributions were calculated with χ^2^ goodness-of-fit tests. Contrasts between variables were performed using χ^2^ contingency analysis. When the assumptions for contingency analysis were not met, permutation independence tests were used as an alternative. In this test, the test statistic and the corresponding *p*-value are estimated by generating all the possible value rearrangements of two variables.

The Bonferroni correction was applied to account for multiple comparisons. The significance level (α = 0.05) was divided by the total number of tests (m), according to α = 0.05/m yielding a new significance threshold reported in connection to all tests. *p*-values were considered significant if they were less than the adjusted significance level.

To investigate how cats utilized available space, a χ^2^ contingency analysis was used to test the effect of cat stay period and customer occupancy on vertical level choice and cat behavior. The expected preference for higher vertical levels was tested using a χ^2^ goodness-of-fit test. Individual differences in behavior was examined with permutation independence tests.

To evaluate the occurrence of cat–cat interaction, the interaction rate was calculated. Cat–cat interactions classified as ambiguous were not considered in the analysis. Behaviors under this category could be interpreted as either affiliative or agonistic depending on the situation, which complicates their objective measurement and analysis. A χ^2^ goodness-of-fit was used to determine whether affiliative interactions were more common than agonistic. Additionally, the effect of a cat’s individuality on interaction type was tested using a permutation independence test. The effect of cat sex was examined using χ^2^ contingency analysis.

The variables human age and gender were combined to create separate human subject groups (i.e., boys, girls, women, and men), and some behaviors were selected for analysis (i.e., approach cat, approach person, play, stroke cat, active avoidance, passive avoidance, and talk to cat), as similarly described in the literature [37,38]. Intraspecific interactions with the staff were excluded from the analysis during this stage of the study. The effect of the human group (i.e., age and gender) on the interactions with cats was evaluated with χ^2^ contingency analysis and an association plot of Pearson residuals indicating where observed and expected frequencies differ within a contingency table.

A Mann–Whitney U test was performed to examine potential differences between bookings (expected number of visitors for the full day) on weekdays compared to weekends. Three observation days, 1 weekday and 2 weekend days, contained missing values and were excluded from the analysis.

## 3. Results

**Figure 2:**
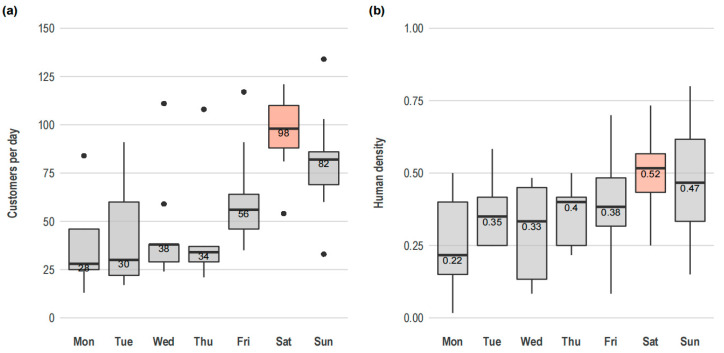
Daily bookings and human density in the cat café. Graphs show median, interquartile range, and total range of (**a**) customers per day and (**b**) human density (customers/m^2^). The groups with the highest median values are highlighted in color. Data from 107 observations.

Over the course of the study, a median of 59 customers per day (IQR 33–89.5; min = 13; max = 134) visited the cat café. The median human density per observation was 0.42 customers/m^2^ (IQR 0.32–0.53; min = 0.02; max = 0.80). Both the number of customers per day and human density were higher during the weekends, especially on Saturdays (Figure 2). Weekend bookings (n = 26, mdn = 84.5, mean rank = 95.81) were significantly more numerous than weekday bookings (n = 41, mdn = 34, mean rank = 43.59) (*U* (41, 26) = 212.00, z = −5.53, *p* < 0.001, r = 0.68, indicating a large effect size).

### 3.1. Space Use

**Figure 3:**
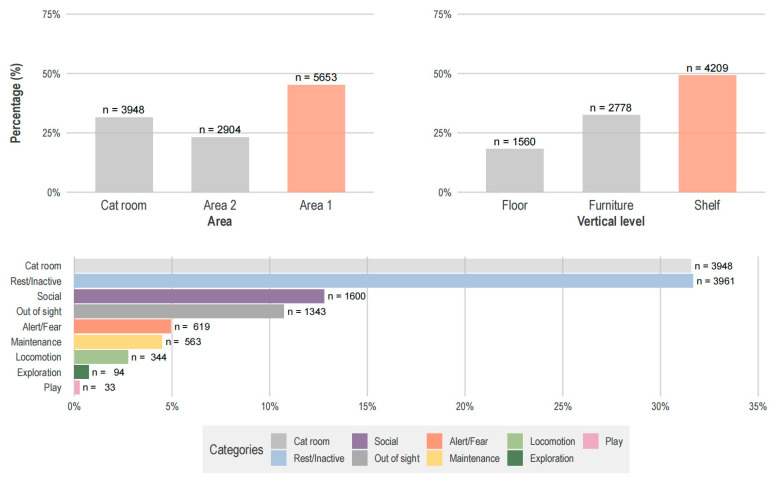
Space use and behavior in the cat café. Graphs show the number of observations (n) and proportions (%). Data from 107 observations (12,505 scans).

Cats spent 31.6% of the group scans in the cat room and more than half in the lounge (68.4%), particularly in area 1 (45.2%) (Figure 3). Although there were individual differences in vertical level choice (χ^2^ permutation test, χ^2^ = 2800.896, *p* < 0.001, post hoc Bonferroni correction *p*_adj_ = 0.013), the preference for higher vertical levels was clear among cats (49.3%) (χ^2^-goodness-of-fit test, χ^2^ (2) = 1234.2, *p* < 0.001, post hoc Bonferroni correction *p*_adj_ = 0.013).

**Figure 4:**
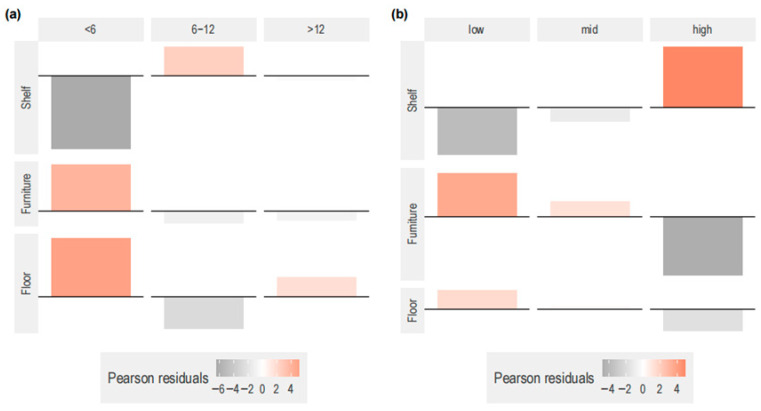
Effect of stay period and customer occupancy on vertical level choice. The association plots represent the Pearson residuals for the combination of (**a**) vertical level choice and stay period (weeks) and (**b**) vertical level choice and customer occupancy (low: <21 customers, mid: 21–29 customers, high: >29 customers). Data from 107 observations.

**Figure 5:**
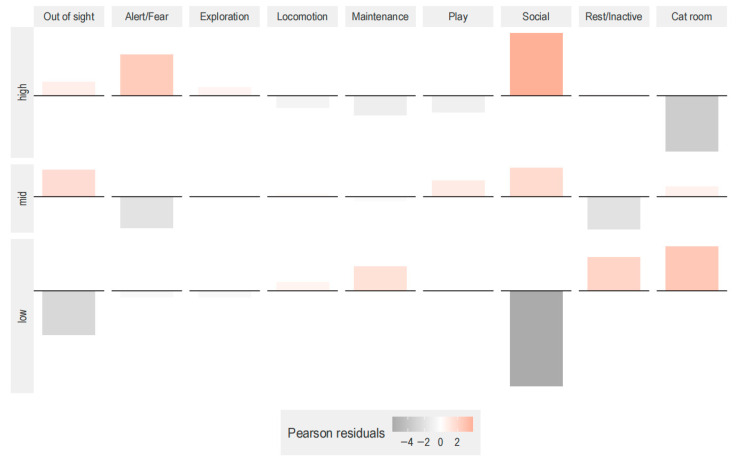
Effect of customer occupancy on cat behavior. The association plot represents the Pearson residuals for the combination of behavioral category and customer occupancy (low: <21 customers, mid: 21–29 customers, high: >29 customers). Data from 107 observations.

The use of vertical space differed in cats depending on stay time in the café (χ^2^ contingency test, χ^2^ (4) = 102.61, *p* < 0.001, also after post hoc Bonferroni correction *p*_adj_ = 0.013). Cats that stayed less than 6 weeks used lower vertical levels (i.e., floor and furniture) more than expected. Preference for higher vertical levels (i.e., shelves) was overrepresented in cats that stayed between 6 and 12 weeks. The use of the floor was slightly overrepresented in cats that stayed more than 12 weeks (Figure 4). Cats utilized elevated vertical levels when customer occupancy was higher (χ^2^ contingency test, χ^2^ (4) = 82.386, *p* < 0.001, *p*_adj_ = 0.013 with post hoc Bonferroni correction), whereas floor and furniture were overrepresented at low-mid customer occupancy (Figure 5).

### 3.2. Behavior

Each cat displayed an individual behavioral repertoire. While in the lounge, resting was the most registered behavior (31.7%), followed by social behaviors (12.8%) and being out of sight (10.7%) (Figure 3).

The behavioral repertoire of the cats differed depending on stay time in the cat café (χ^2^ contingency test, χ^2^ (16) = 1113.2, *p* < 0.001, *p*_adj_ < 0.025 after post hoc Bonferroni correction). Cats engaged more than expected in play, rest, maintenance, or being absent from the lounge when there were fewer than 29 visitors in the cat café over the course of the observation session (i.e., low and mid customer occupancy) (Figure 5).

### 3.3. Intraspecific Interactions

Three observations did not include any social interactions and were excluded from analyses. The rate of cat–cat interactions was 0.58 interactions/cat/h (n = 349 interactions). Affiliative interactions were calculated at 53% while agonistic at 47.0%, but this difference in frequency was not statistically significant (χ^2^ goodness-of-fit test, χ^2^ (1) = 1.264, *p* = 0.261, *p*_adj_ < 0.017 after post hoc Bonferroni correction). The type of intraspecific interaction depended on the initiator (χ^2^ permutation test, χ^2^ =96.964, *p* < 0.001, *p*_adj_ < 0.017 after post hoc Bonferroni correction), in that the cats displayed individual variation in their affiliative and agonistic behaviors. However, the sex of the interacting cats did not influence the type of interaction in our sample (χ^2^ contingency test, χ^2^ (3) = 3.343, *p* = 0.342, *p*_adj_ < 0.017 after post hoc Bonferroni correction).

### 3.4. Customer–Cat Interactions

During 44.4% of the focal observation time, there were no cat–customer interactions. Of the interactions observed, behaviors under the non-contact attention category were registered more often (29.0%) than the categories for contact attention (23.2%) and no attention (3.4%). The most frequent human behavior registered was stroking a cat (19.2%), followed by approaching a cat (16.1%).

**Figure 6:**
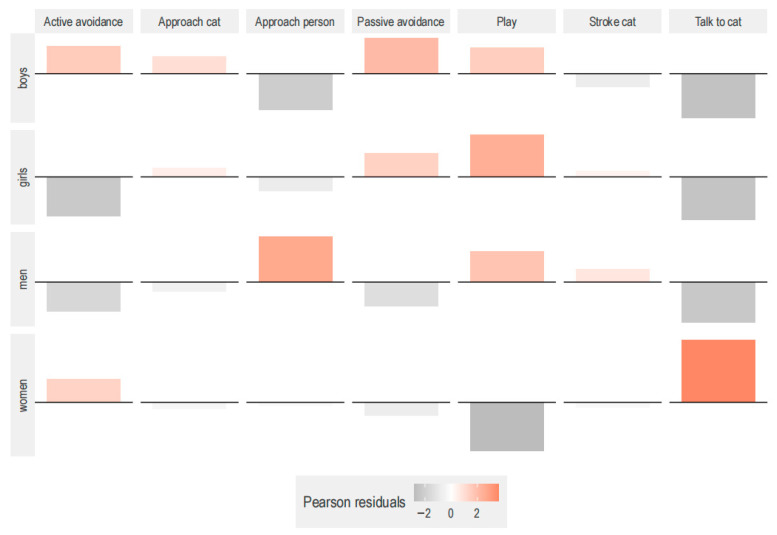
Effect of human age and gender on human–cat interactions. The association plot represents the Pearson residuals for the combination of behaviors of interest and human composition (boys, girls, men, and women). The number of interactions per human group were boys (332), girls (447), men (620), and women (1911). Data from 70 observations. The human–cat interactions were significantly influenced by human age and gender (χ^2^ contingency test, χ^2^ (16) = 107.07, *p* < 0.001).

Among children and men, the third most registered behavior was play (boys: 9.9%; girls: 10.3%; men: 9.0%), whereas this was represented in women by talk to cat (8.9%). Most human–cat interactions occurred in area 1 (75.9% vs. 24.1%) and were mainly initiated by the customers (16.1% vs. 2.6%). Human–cat interaction was significantly influenced by human age and gender (χ^2^ contingency test, χ^2^ (18) = 87.416, *p* < 0.001) (Figure 6). Looking at the effect of human age and gender on human–cat interactions (Figure 6), children approached cats more than expected. Passive avoidance in cats was found overrepresented when interacting with children, although active avoidance was observed more than expected towards boys and women. Play was more common than expected when interacting with girls, boys, and men, but no great differences in petting were found among human groups. Cats also approached men more than expected and children less than expected. Women talked or made non-verbal sounds to the cats (i.e., vocalized) significantly more than expected.

## 4. Discussion

We identified that cats’ use of vertical space increased during periods of higher customer occupancy as well as in cats having spent a longer time in the café. Customer–cat interactions were primarily initiated by the customer and were not present during 44% of the focal observations. Despite the attentive care of the cat café staff, there were some indications of behaviors related to stress among the cats, such as hiding and decreased maintenance behaviors.

Results indicated a preference for elevated vertical levels and concealed spots, especially when customer occupancy was high. Previous studies show that hiding and perching are essential behaviors for domestic cats as part of the coping mechanisms when facing new environments [26,30] and potential stressors. Domestic cats still prioritize the security of their territory over relationships with conspecifics due to their evolutionary origin as small prey animals [51]. Elevated areas may create a sense of control in the cats that contribute to coping in an unpredictable environment like the cat café, where customers and conspecifics are changing and unfamiliar [52].

Surprisingly, resting and inactivity were more common than expected during lower customer occupancy. These findings suggest that the possibility of rest could have been disturbed by customers when the occupation levels were higher in the café, and that the cats therefore compensated for this. This is supported by the cats’ increased use of the vertical level shelf during this period, as well as the rise in cat–customer interactions with higher occupancy. Previous research shows that humans are usually the ones to initiate interaction during first encounters with cats [37], so more customers are probably leading to more human-initiated contact. Since interaction time was limited, usually one or two hours, customers could have been particularly motivated to follow this predisposition, especially if there were more humans in the room and the aim of their visit to the cat café was to meet and interact with the cats. Similarly, the cats used the cat room (only available to cats and staff) more than expected at lower customer occupancy, possibly due to the ability to move around more freely during lower human densities, as cats avoid conflict by keeping distance [18,21].

In line with our expectations, maintenance behaviors were underrepresented when customer occupancy was high. This finding is consistent with that of Rehnberg et al. [27], who found a positive association between suppressed maintenance behaviors and behavioral stress in owned cats facing a new environment. The absence of these normal cat behaviors has been seen in shelter cats rated as frustrated and was also connected to the cats’ immune function [10]. Similarly, locomotion, exploration, and play behaviors represented a small proportion of the behavioral repertoire of the study cats, and play was suppressed when customer occupancy was high. Chronically stressed cats from a lab facility showed suppressed exploratory and play behavior when exposed to unpredictable handling by humans [26]. Play has been defined as a good welfare indicator in previous research [53] and could be a sign that other behavioral needs are fulfilled.

When looking at intraspecific interactions, these were infrequent at the cat café. These results are consistent with data obtained by Hirsch et al. [54], who found less than 0.5 interactions per cat/h in a similar setting, where shelter cats were exposed to unstable cat groups and frequent contact with visitors. Affiliative behaviors occurred approximately in the same proportion as agonistic, and, contrary to our expectations, we observed few interactions between the three related groups of cats or five pairs of previously familiar cats in the study. This finding is not supported by previous studies, which have suggested that familiarity and relatedness are positively associated with increased proximity and affiliative behaviors [23] as well as guardians’ rating cats as having a positive relationship [55]. A possible explanation for these results might be the constant flux of cats entering or leaving (adopted from) the cat café, a situation which could have disturbed the social relationships within the group [19]. In the present study, cats avoided other individuals by maintaining distance or concealing their presence. In a study examining different housing conditions for shelter cats, aggression levels were reported to be higher when cats were unable to avoid other individuals [18]. The large proportion of resting and inactive behaviors observed in our study could provide further support for the cats avoiding other individuals at the cat café. Some studies argue that decreased activity levels are also a consequence of cats establishing preferred spots [29,45], which is also consistent with the observer’s perception in this study. However, the current methodology was not designed to discriminate between the uses of different specific resources (e.g., cat trees, hiding places, etc.) in the cat café.

The observed cats displayed great individual variation in their interactions towards conspecifics, but there was no significant difference that could be attributed to the sex of the cat. These results corroborate the findings from Barry and Crowell-Davis [22], who observed little effect of cat sex on the social (affiliative/agonistic) behavior of indoor, neutered cats in private homes, even if male dyads spent more time in closer proximity compared to other combinations. Still, these results conflict with a recent study showing that guardians of spayed female dyads were more likely to report a negative relationship [55]. The background of the cats may have contributed to the observed individual variation. Most of the cats were former pets with a median age of four years. However, the socialization status was uncertain for some cats due to the lack of information about their previous life. Earlier studies have noted that formerly owned cats of similar ages showed higher behavioral stress when entering a shelter compared to formerly stray cats [28], and non-socialized individuals can increase the behavioral stress signs within a group [20]. Further research is required to establish the effect of age, socialization status, and origin on cat–cat interactions during housing in cat cafés and to provide general guidelines to integrate new individuals into preexisting groups.

Our second aim was to observe the human–cat interactions at the cat café. Surprisingly, no human–cat interactions occurred during almost half of the focal observation time. The high number of customers and human density observed might have influenced this. Research indicates that parameters such as human density and unpredictable handling elicit chronic physiological stress in domestic cats, which may be expressed as inactivity and withdrawal behavior [32]. The cats spent more than half of the observation time engaged in resting behaviors or absent from the cat café lounge. However, a potential limitation of the present methodology was the challenge to distinguish between true and feigned sleep, which has been suggested as a behavior indicative of stress in previous studies [56,57]. Further research should investigate the potential effect of feigned sleep in relation to human-related parameters at the cat café.

Few studies have examined how cats interact with humans, especially research published in English. However, comparing the current findings with previous studies confirms that the cats reacted to differences in human behavior in relation to age and gender. Consistent with the literature, customers usually initiated interactions with cats, especially children [37], who also tended to follow the café cats after withdrawal. The data suggest that the study cats remained passive to interactions with youth in general but reacted actively to boys and women by walking away or dodging touch. Research indicates that cats prefer adults over children and show more approach behavior towards females, possibly due to their increased presence and role as primary feeder at home [38]. This is consistent with the current results, since most interactions occurred between adult females and cats, and female customers were more common. Whereas the amount of stroking and play with cats is similar among human groups during first encounters [37], female adults played less with the study cats. Instead, our findings suggest that women vocalized with the cats significantly more than other visitors at the cat café, which is in line with data obtained in previous studies [37,38]. Playing and stroking may have a positive influence on socialized café cats, since research shows that these are the preferred interactions with humans [12]. Future studies should also address the potential positive effects of consistent interactions with the cat café staff, since interactions with familiar humans can improve the welfare and health of shelter cats [10]. In contrast to the report by Mertens & Turner [37], the study cats approached men more than other customers at the cat café. These results are likely to be related to differences in human activity and/or posture that could have influenced the cats’ willingness to approach. Although some reports found significant differences in body posture among human groups [37], further research is needed to confirm this in the present setting. In addition, future studies should consider the duration of human–cat interactions at the cat café and improve the definition of age groups among the customers (i.e., young adults, elderly, etc.). The higher number of booked customers during weekends compared to weekdays was expected. However, this pattern may indicate a need for additional opportunities for rest or modified opening hours to maintain optimal cat welfare. The use of booking data (total expected visitors per day) rather than the actual number of visitors present during observations was chosen because cats are affected by the cumulative exposure to unfamiliar individuals throughout the day, not only those present during the specific observation periods. Since cats are known to require time to recover after stressful events [58], booking numbers likely provide a more accurate representation of potential stressors from customers, particularly given that no more than 14 customers were allowed at any given time.

There were two primary limitations to the study. The first is a lack of inter- and intra-observer reliability. As the observations took place at a business during opening hours, we could not videorecord observations for reliability testing. Due to restrictions related to the COVID-19 pandemic, we could not include two observers at the same time. A further limitation to the generalization of these results is the impact of restrictions related to the COVID-19 pandemic on the study site. The circumstances led to several feeding and schedule changes that might have influenced cat behavior, as well as regulations in the customers’ entrance. Our findings should be considered under the unique physical and social conditions of the cat café, and, therefore, results should be extrapolated to other cat cafés and group housing situations with care. There were also several factors out of our control, since the study site was an applied setting. Some customers attempted to reach cats resting on higher shelves or draw the attention of cats that had retreated to the cat room. However, research shows that this could be self-defeating when interacting with cats, since the more successful a person is at initiating an interaction, the shorter the interaction is [39]. Thus, the staff should further remind customers that all interactions should occur on the cats’ terms to achieve the best experience at the cat café.

This study highlights the need for more research focused on the welfare of cats in cat cafés to provide general guidelines for improving their living conditions. An important but unanswered question is how changes in behavior over the cats’ stay will affect their performance in the cat café’s physical and social environment. The current study only provides a snapshot of the behavioral repertoire of cats that stayed less than 6 weeks, between 6 and 12 weeks, and more than 12 weeks in the cat café. Additional research is needed to understand how the increased levels of inactivity and absence from the lounge observed in this study are affected by time and relate to cat stress, health, and social interactions with conspecifics and customers.

## 5. Conclusions

To our knowledge, the present study is the first to examine the physical and social environment in a cat café, focusing on cats from an ethological perspective. The results of our study identified the importance of elevated structures for domestic cats housed in cafés, as we found that higher customer occupancy was associated with a preference for elevated vertical levels. This indicates that the physical and social environment needs to be planned and organized to achieve good welfare for the café cats. Thus, we suggest that providing complex vertical space and hiding spots in cat cafés is necessary, as it will promote cat welfare and natural behaviors, and it may improve group stability by allowing cats to avoid other individuals. The layout of cat cafés should also include a private cat area since the use of the cat room in this study, which was only accessible to cats, was influenced by customer occupancy. Cats are subjected to large numbers of humans during their stay at the cat café before adoption. If and how this environment affects the cats’ future relationships with humans should be further investigated. In future studies, it might be possible to focus more on the importance of exploration and play behavior on the welfare of cats in the cat café setting. More research on the effect of cat background on intraspecific interactions at the cat café will contribute to the understanding of how to include new individuals into preexisting groups. Adjusting the number of customers allowed or promoting adoption will also enhance the welfare of café cats, since high human density and unpredictable handling elicit chronic stress [26,32] and there is a higher risk of infectious disease in cats that stay for extended periods at shelters [18]. Further research is needed on the understanding of human–cat interactions in the cat café and its potential effects on the visitors.

## Figures and Tables

**Table 1:** Demographics of the 27 cats included in the study.

Name	Sex	Age (Years)	Color	Origin	Socialization Status		Stay (Weeks)
					Humans	Conspecifics	
Aramis	M	6	Brown tabby	S	Uncertain	Uncertain	10
Sune	M	4	Orange bicolor	H	Yes	Yes	10, 10 *
Snobben	M	4	Orange bicolor	H	Yes	Yes	19
Tequila	F	5	Brown bicolor	H	Yes	Yes	8
Nemo	M	4	Black bicolor	H	Yes	Yes	8
My	F	4	Diluted tricolor	C	Uncertain	Yes	14
Humlan	F	<1	Brown bicolor	S	Uncertain	Yes	6
Hedda	F	<1	Brown bicolor	S	Uncertain	Yes	6
Gustav	M	7	Orange bicolor	H	Yes	Yes	17
Zimmerman	M	3	Orange bicolor	H	Yes	Uncertain	9
Emma	F	2	Tricolor	S	Uncertain	Uncertain	**14**
Scarlett	F	10	Gray (BSH **)	H	Yes	Yes	8
Jack	M	5	Gray bicolor (BSH)	H	Yes	Yes	8
Sockan	F	6	Gray	H	Yes	Yes	8
Tussan	F	9	Tricolor	H	Yes	Yes	6
Dextra	F	1	Black bicolor	H	Uncertain	Uncertain	12
Nisse	M	1	Orange bicolor	H	Yes	Yes	8
Tösen	F	1	Brown tabby	H	Yes	Yes	8
Alba	F	4	Brown tabby	S	Uncertain	Yes	1
Ruben	M	8	Black bicolor	H	Yes	Uncertain	**10**
Elvis	M	<1	Ticked gray	C	Uncertain	Yes	**8**
Teddy	M	<1	Shaded gray	S	Uncertain	Uncertain	**8**
Louie	M	6	Orange bicolor	H	Yes	Yes	7
Phoebe	F	1	Orange bicolor	H	Yes	Yes	7
Cissi	F	<1	Gray	H	Yes	Uncertain	**3**
Lisa	F	<1	Bengal mix	H	Yes	Uncertain	**3**
Luna	F	4	Black bicolor	H	Yes	Uncertain	**2**

* Double stay: The cat was adopted and returned during the course of the study. ** BSH: British shorthair. NOTE: Individuals are arranged according to their arrival. Coat color was identified with the guide from [42]. The breed of pedigree cats is in parentheses. Origin is abbreviated as: stray (S), home (H), or colony (C). Numbers in bold represent the stay period of cats that remained beyond completion of observations.

## Data Availability

The raw data supporting the conclusions of this article will be made available by the authors on request.

## Supplementary Materials

The following supporting information can be downloaded at: https://www.mdpi.com/article/10.3390/ani15223233/s1, Table S1: Ethogram of behaviors using scan sampling; Table S2: Ethogram of cat–cat interactions using all-occurrences sampling; Table S3: Ethogram of social behaviors during human–cat interactions using continuous sampling.
