# Supplementary material for: Cats in a Cat Café: Individual Cat Behavior and Interactions with Humans
Source: Animals (Basel). 2025 Nov 7;15(22):3233. doi: 10.3390/ani15223233 (PMC12649627; doi:10.3390/ani15223233)
Supplement: Supplementary file 1 [file animals-15-03233-s001.zip › animals-3887237-supplementary.pdf]

Table S1. Ethogram of behaviors for scan sampling.

Category	Behavior	Definition
Maintenance	Drink	Cat ingests water (or other liquids) by lapping up with the tongue.
	Eat	Cat ingests food by means of chewing with the teeth and swallowing.
	Groom	Cat cleans itself by licking, scratching, biting, or chewing the fur on its body. May also include the licking of a front paw and wiping it over one's head.
	Scratching	Cat drags front claws along an object or surface, likely leaving visual marks behind.
	Urinate	Cat releases urine on the ground while in a squatting position.
	Defecate	Cat releases feces on the ground while in a squatting position.
Rest/inactive	Lying	Cat's body is on the ground in a horizontal position, including on its side, back, belly, or curled in a circular formation.
	Sitting	Cat is in an upright position, with the hind legs flexed and resting on the ground, while front legs are extended and straight.
	Sleeping	Cat is lying on the ground with its head down and eyes closed, performing minimal head or leg movement, and is not easily disturbed.
	Stretching	Cat extends its forelegs while curving its back inwards.
Exploration	Explore	Cat moves around attentively while sniffing the ground and/or objects.
	Flehmen	Cat makes a grimaced facial expression, where the mouth is open, upper lip is elevated, and tongue may protrude out of the mouth. Generally follows the sniffing of an object, scent, bodily excretion, or another cat.
Locomotion	Climb	Cat ascends and/or descends an object or structure.
	Jumping	Cat leaps from one point to another, either vertically or horizontally.
	Walking	Forward locomotion at a slow gait.
	Running	Forward locomotion in a rapid gait, which is faster than walking.
Alert/Fear	Hiding	Cat occupies a location away from a specific stimulus. This can include behind items, under furniture or inside shelters.
	Crouch	Cat is alert and positions the body close to the ground, whereby all four legs are bent, and the belly is touching (or raised slightly off) the ground.
Playing	Solitary play	Cat interacts with something in a "non-serious" manner (i.e. where there is no intention to harm).
	Social play	Cat interacts with a conspecific in a "non-serious" manner (i.e. where there is no intention to harm).
Reproductive	Mount	Cat mounts another cat.
Stereotypic	Fur-plucking	Cat excessively grooms a specific area of its body. May result in the removal and visible loss of fur, as well as skin irritation.
	Pacing	Repetitive locomotion in a fixed pattern, such as back and forth along the same route or circling. Movement seems to have no apparent goal or function. Must be performed at least two times in succession before qualifying as stereotypic.

Social behavior	Cat-cat interaction	Includes any kind of social interaction between cats such as:<br>- Affiliative behaviors and vocalizations.<br>- Agonistic behaviors and vocalizations.<br>- Ambiguous behaviors and vocalizations.
	Human-cat interaction	Includes any kind of social interaction between a cat and a human, such as:<br>- Contact attention.<br>- Non-contact attention (includes behaviors and vocalizations).<br>- No attention.
Out of sight	Out of sight	Cat is not visible to the observer in the lounge.
	Cat room	Cat is not visible to the observer because it is in the cat room (modifier).

Table S2. Ethogram of cat-cat interactions using all-occurrences sampling.

Category	Behavior	Definition
Affiliative	Relaxed approach	Visible to each other, ears directed forward, tail relaxed down or straight up, straight back line.
	Sniff	Cat smells another cat by inhaling air through the nose.
	Head rub	Head moved with contact with other cat's head or body
	Body rub	Body moved with contact with other cat's head or body.
	Social play	Two cats play with the same toy simultaneously or within 3 seconds.
	Allogroom	Cat licks the fur of another cat's head or body.
	Huddling	Cat is at rest, lying or sitting with body in contact with another cat.
	Purr	A purring sound is heard.
	Chirp	A short, high-pitched call is described like a bird chirping. Often used to greet or when something is desired.
Agonistic	Aggressive approach	Visible to each other, ears up directed sideways, tail down, piloerection, stiff legs, back line higher towards the tail.
	Afraid approach	Visible to each other, ears partly or totally pressed to the head, lowered head, back line lowered or curved, often crouched walking, tail between legs often with piloerection.
	Crouch	Ears partly or totally pressed to the head, lowered head, back line lowered or curved, often crouching, withdraws, tail between legs often with piloerection.
	Avoid	Cat moves away from other cat who has initiated contact, or changes direction when passing.
	Strikes air	Cat strikes with the paw towards other cat without hitting, one time or several times in a row. No toy involved.
	Strikes cat	Cat strikes with the paw and hits other cat, one time or several times in a row. No toy involved.
	Intense stare	Cat gazes fixedly at another cat.

	Avoid eye contact	Cat avoids eye contact with another cat, by averting the eyes or turning the head or the body away.
	Bite	Cat bites another cat.
	Hiss	A hissing sound is heard.
	Howl	Tonal sound, typically shorter than yowls. Usually agonistic or defensive.
	Growl	A deep burring sound is heard. Mainly used to signal danger or to warn or scare off an opponent.
	Spit	Cat makes a sudden, short, explosive exhalation often accompanied by a violent movement.
	Pain shriek	Short intense cries that are characterized by great strain at mouth and throat and the force of breath. Often heard in active fighting.
Ambiguous	Wrestle	Cats lying on the side, with front paws round each other, can kick with back paws and bite.
	Attack	Cat jumps on other cat or makes a lunge without contact.
	Chase	Cat runs after other cat without contact.
	Run	Cat runs away from other cat without contact.
	Roll up	Cat rolls onto its back with its belly exposed and all paws in the air.
	Meow	The distinctive "meow" call is heard. Meowing has attention seeking properties and its valence can differ between situations.

Table S3. Ethogram of social behaviors during human-cat interactions using continuous sampling.

Category	Behavior	Definition
Contact attention	Head butt	Cat briefly pushes its head against person.
	Kneading	Cat pushes forepaws towards the person's body in a rhythmic, kneading motion. Claws may be extended, retracted, or both (alternating).
	Body rub	Cat rubs any part or entire length of body against a person.
	Stroke cat	Person strokes any part or entire body of the cat.
	Lick	Cat's tongue protrudes from mouth and contact a part of the person's body.
	Sniff	Cat smells person by inhaling air through the nose.
	Strike at person	Cat aims to strike at a person with the paw, one or several times in a row. No toy involved.
	Rest	Cat is at rest, lying or sitting with body in contact or within 40 cm of a person.
Non-contact attention	Approach cat	Person moves to within-reach distance of the cat.
	Approach person	Cat moves to within-reach distance of the person.
	Tail up	Tail is held in an upright position and the cat may approach the person.
	Staring	Cat gazes fixedly at person and is not easily distracted.
	Play	A cat and a person play with a toy or the cat interacts with the person with no intention to harm.
	Meow	The distinctive "meow" call typical of cats is heard while interacting with a person.
	Purr	A purring sound is heard.
	Chirp	A short, high-pitched call is heard while interacting with a person. Often used to greet or when something is desired.
	Hiss	A hissing sound is heard while interacting with a person.
	Growl	A deep burring sound is heard while interacting with a person.
	Howl	Tonal sound, typically shorter than yowls. Usually agonistic or defensive.
	Talk to cat	Person speaks to cat or makes non-verbal sounds to cat.
No attention	Active avoidance	The cat avoids a person who has initiated contact, either by walking away or dodging touch.
	Passive avoidance	The cat remains passive towards a person who has initiated contact. It may precede a negative interaction.
No interaction	No interaction	The cat is not performing any kind of interaction with a person.
